# Journals

**Published:** 1881-03

**Authors:** 


### BIBLIOGRAPHICAL.

*Journals.*—

Volume II of the *Independent Practitioner* comes to hand, thus entering on the second year of its existence. This is a Baltimore enterprise, and is spicily edited by Harvey L. Byrd, A. M., M. D., and Basil M. Wilkerson, D. D. S., M. D., at 68 North Charles Street,—price $2.00 per annum. The theory on which the *Practitioner* is published is, that it shall be an organ of both the medical and dental professions. The number before us, for February, seems entirely devoted to medical science, as we do not notice any papers on dentistry. Our profession at large read little enough of medicine we suspect, and so it may be just as well to give them a chance of finding medical papers in a semi dental journal. The editors have a good deal of enterprise to start a new journal amidst the multitude of quarterlies, monthlies, bi-weeklies, with a threat of a daily sometimes, and the fact that they continue it into the second year is, we suppose, proof that it has life and aim. The editors claim that it "has the largest circulation of any medical periodical this side of Philadelphia," a claim which if correct, is certainly a remarkable success, provided this is a cash and not credit circulation.

